# Acute kidney disease in hospitalized pediatric patients: risk prediction based on an artificial intelligence approach

**DOI:** 10.1080/0886022X.2024.2438858

**Published:** 2024-12-12

**Authors:** Lingyu Xu, Siqi Jiang, Chenyu Li, Xue Gao, Chen Guan, Tianyang Li, Ningxin Zhang, Shuang Gao, Xinyuan Wang, Yanfei Wang, Lin Che, Yan Xu

**Affiliations:** a Department of Nephrology, the Affiliated Hospital of Qingdao University, Qingdao, China; b Division of Nephrology, Medizinische Klinik und Poliklinik IV, Klinikum der Universität, Munich, Germany; c Ocean University of China, Qingdao, China

**Keywords:** Pediatric, acute kidney injury, acute kidney disease, machine learning, prediction model, renal function trajectory

## Abstract

**Background:**

Acute kidney injury (AKI) and acute kidney disease (AKD) are prevalent among pediatric patients, both linked to increased mortality and extended hospital stays. Early detection of kidney injury is crucial for improving outcomes. This study presents a machine learning-based risk prediction model for AKI and AKD in pediatric patients, enabling personalized risk predictions.

**Methods:**

Data from 2,346 hospitalized pediatric patients, collected between January 2020 and January 2023, were divided into an 85% training set and a 15% test set. Predictive models were constructed using eight machine learning algorithms and two ensemble algorithms, with the optimal model identified through AUROC. SHAP was used to interpret the model, and an online prediction tool was developed with Streamlit to predict AKI and AKD.

**Results:**

The incidence of AKI and AKD were 14.90% and 16.26%, respectively. Patients with AKD combined with AKI had the highest mortality rate, at 6.94%, when analyzed by renal function trajectories. The LightGBM algorithm showed superior predictive performance for both AKI and AKD (AUROC: 0.813, 0.744). SHAP identified top predictors for AKI as serum creatinine, white blood cell count, neutrophil count, and lactate dehydrogenase, while key predictors for AKD included proton pump inhibitor, blood glucose, hemoglobin, and AKI grade.

**Conclusion:**

The high incidence of AKI and AKD among hospitalized children warrants attention. Renal function trajectories are strongly associated with prognosis. Supported by a web-based tool, machine learning models can effectively predict AKI and AKD, facilitating early identification of high-risk pediatric patients and potentially improving outcomes.

## Introduction

Acute kidney injury (AKI) is a clinical syndrome marked by a rapid decline in kidney function within hours to days [1]. AKI affects approximately 12% to 40% of pediatric inpatients [2], making it a major complication in this population and is associated with increased mortality, prolonged hospital stays, and a higher risk of progression to chronic kidney disease (CKD) [3–5]. The timely and accurate identification of high-risk pediatric AKI patients, along with prompt treatment, is essential for improving patient outcomes.

In 2017, the Acute Disease Quality Initiative (ADQI) 16 Workgroup proposed that AKI and CKD might not be separate pathophysiological processes but rather represent a continuum in renal dysfunction progression. They introduced the concept of acute kidney disease (AKD) to define an intermediate phase in the transition from AKI to CKD, characterized by persistent renal dysfunction lasting 7 to 90 days after an initial kidney injury event. This period is critical for determining whether renal function can recover; timely intervention during this phase may prevent progression to CKD and improve patient outcomes [6]. Furthermore, ADQI highlighted that the AKI-AKD continuum can progress along different trajectories, each associated with distinct implications for patient prognosis.

Serum creatinine (Scr) and urine output are standard diagnostic indicators for acute and subacute kidney injury [6,7]. However, these markers can only be detected after kidney injury has already occurred and are often influenced by non-renal factors [8], which limits their sensitivity and specificity [9]. New risk prediction models based on electronic health records have gained attention [10], with the 15th ADQI consensus conference emphasizing the significant role of artificial intelligence [11]. Machine learning, a subset of artificial intelligence, identifies complex patterns within multidimensional data, enabling accurate classification of new cases and predictive outputs, often surpassing traditional logistic regression algorithms in accuracy [12].

Existing machine learning risk prediction models for kidney injury in hospitalized children are predominantly focused on AKI associated with intensive care units and cardiac surgery [13–15], leaving a gap in research on AKI within general pediatric wards. Studies have shown that the incidence of AKD can reach up to 52.2% in pediatric intensive care units [16], and 56.3% of AKI survivors in general pediatric wards may progress to AKD [17]. Despite these findings, a comprehensive understanding of AKD epidemiology in hospitalized pediatric patients remains lacking [18]. To date, no machine learning-based risk prediction models have been developed for AKD in general pediatric patients, nor have any studies examined adverse outcomes in this population based on renal function trajectories.

This study aims to: (a) to explore the incidence and mortality of AKI, AKD, and different renal function trajectories along the AKI-AKD continuum; (b) construct a risk prediction model for AKI and AKD in general pediatric patients, assessing its predictive performance through 10-fold cross-validation and a test dataset; and (c) develop an online tool to facilitate early identification of children at high risk for kidney injury, enabling healthcare providers to make timely, risk-informed decisions.

## Materials and methods

### Study cohort

This retrospective study analyzed 2,346 pediatric inpatients from a tertiary healthcare institution in China, all of whom had at least two Scr measurements taken between January 2020 and January 2023. The exclusion criteria were as follows: (a) patients younger than 28 days (neonates) or older than 18 years; (b) hospital stays shorter than 24 h; and (c) patients with a history of kidney transplantation, any form of dialysis, or preexisting end-stage renal disease, defined as an estimated glomerular filtration rate (eGFR) of ≤ 15 mL/min/1.73 m^2^ prior to admission.

### Ethical approval

The study was approved by the Ethics Committee of The Affiliated Hospital of Qingdao University (IRB: QYFYWZLL28554). Clinical data were extracted from electronic medical records and anonymized to ensure patient confidentiality. The anonymization process was strictly supervised by the Ethics Committee, including replacing names with identification codes prior to analysis and removing private information. As the study posed minimal risk to participants, informed consent was not required.

### Data collection

Available variables, including patient demographics, laboratory tests, comorbidities, and concomitant medications, were collected from electronic medical records. Demographic characteristics encompassed gender, age, body mass index, blood pressure, and hospitalization-related factors, such as length of stay and surgery. A complete blood count, coagulation markers, blood chemistry analysis, and urinalysis were all conducted within the first 3 days after admission, with the initial values recorded being used for analysis. Physiological parameters—including blood glucose, heart rate, respiratory rate, systolic and diastolic blood pressure, body temperature, height, and weight—were measured upon admission, with the first recorded values applied. Comorbidities, such as hypertension and diabetes, were defined according to the ICD-10 Code. Information on the utilization of medications, including hormones, antibiotics, and proton pump inhibitors (PPI), was collected. The eGFR for all pediatric patients was estimated using the modified Schwartz equation (2009) [19].

### Outcome definitions

We considered the occurrence of AKI or AKD during the hospitalization as the outcomes. AKI was diagnosed according to the 2012 Kidney Disease: clinical practice guidelines for improving global outcomes (KDIGO) guidelines, defined as an increase in scr level of > 26.5 μmol/L (0.3 mg/dL) within 48 h, or an increase in Scr level to > 1.5 times the baseline within 7 days [7]. The staging criteria for AKI are defined as follows: Stage 1 is characterized by an increase in serum creatinine to 1.5 to 1.9 times the baseline value, or an absolute increase of ≥0.3 mg/dL (≥26.5 µmol/L). Stage 2 is defined by an increase in serum creatinine to 2.0 to 2.9 times the baseline. Stage 3 is characterized by an increase in serum creatinine to 3.0 times the baseline value, a serum creatinine level of ≥4.0 mg/dL (≥353.6 μmol/L), a decrease in eGFR to <35 mL/min/1.73 m^2^ for patients under 18 years of age, or the initiation of renal replacement therapy [20]. According to the 2017 ADQI definition, the staging criteria for AKD are analogous to those for AKI, with the primary difference being the time frame, which spans from 7 to 90 days following the occurrence of a kidney injury event [6]. The baseline Scr was defined as the first Scr level measured within 3 days after admission. The diagnosis time is established when the patient’s clinical manifestations or laboratory test results first meet the diagnostic criteria for AKI or AKD.

We classified renal function trajectories into three categories based on the ADQI definition [6]: (a) AKI recovery, where renal deterioration is limited to within 7 days; (b) subacute AKD, where renal function gradually declines within the first 7 days without meeting AKI diagnostic criteria, but subsequently progresses to AKD; (c) AKD with AKI, where renal function deteriorates to meet AKI criteria within 7 days and progresses to AKD after 7 days. Patients were ultimately classified into four groups: no kidney disease (NKD), AKI recovery, subacute AKD, and AKD with AKI.

### Model development and performance evaluation

The dataset was randomly split into 85% for the training set and 15% for the test set. The training set was used to develop the model, while the test set was reserved for evaluating its performance. Eight machine learning algorithms were employed to construct the model: Light Gradient Boosting Machine (LightGBM), Gradient Boosting Machine (GBM), Naive Bayes (NB) classifier, Random Forest (RF), K-Nearest Neighbors (KNN), Support Vector Machines (SVM), Multilayer Perceptron Neural Network (MLP-NN), and Logistic Regression (LR). Grid search with 10-fold cross-validation was utilized to optimize model parameters [21].

LightGBM and GBM are gradient boosting frameworks that utilize decision tree algorithms. LightGBM surpasses GBM in training speed, efficiency, memory conservation, accuracy enhancement, scalability, and compatibility with parallel and GPU systems [22]. The NB algorithm computes the posterior probability of a category given a feature condition, based on Bayes’ theorem [23]. RF employs randomization to produce multiple decision trees, which aggregate predictions through voting for classification tasks or averaging for regression tasks, respectively [24]. KNN diverges from NB by eschewing probability calculations, offering a simpler methodological approach [25]. SVM identifies a hyperplane to separate data into two classes, maximizing margin while minimizing classification errors [26]. MLP-NN mirrors the neural network structure of the brain, comprising interconnected nodes where the output of one node serves as input for another node in the processing [27]. LR is an extension of standard regression, customized for modeling binary variables indicating event presence or absence [28].

Additionally, we constructed an ensemble model comprising eight machine learning algorithms as base learners. By integrating predictions from these diverse models, we aimed to reduce the predictive bias inherent in individual models. Both voting and stacking approaches were employed for model integration [29]. ‘Soft Voting’ generates the final prediction by taking a weighted average of the predicted probabilities for each class across all base models, effectively leveraging each model’s confidence level according to its assigned weight [30]. In contrast, ‘Stacking’ combines the predictions of base models through a meta-learner, which uses the output predictions of each base model for each sample as input, enhancing the ensemble’s overall performance [31]. To evaluate model performance, each of the eight machine learning algorithms was alternately designated as the meta-learner, with the remaining seven serving as base models. Each base model was configured using the same hyperparameters as in their individual machine learning model.

Calculate the area under the receiver operating characteristic curve (AUROC), precision, recall, accuracy, F1 score, Brier score, and Matthews correlation coefficient (MCC) to evaluate the model’s performance. The best model for further analysis was selected by comparing AUROC. Decision curve analysis (DCA) was used to assess the clinical effectiveness of the model [32].

### Model explanation

Machine learning outputs are frequently challenged by the ‘black box’ problem, underscoring the need for accurate interpretation of model predictions. To address this, we applied SHapley Additive exPlanations (SHAP) values, which evaluate the contribution of each variable and provide interpretability from both global and local perspectives. SHAP offers a unified framework for explaining machine learning models by delivering consistent and locally precise attribution values for each feature [33]. In our study, SHAP summary plots and bee swarm plots were used for a comprehensive overview, while force plots and decision plots facilitated the interpretation of individual predictions. SHAP interaction and dependence plots further highlighted the relationships between different features.

### Data rebalancing

We encountered data imbalance with AKI and AKD incidences of 14.8% and 16.2%, respectively. To mitigate this issue, we employed weight rebalancing techniques to adjust class weights during model training. To maintain balance in the NB classifier, we standardized prior probabilities to 0.5 for each category. For the MLP, we adjusted class weights within the loss function to effectively tackle the imbalance. Utilizing the ‘class_weight’ parameter in LR, RF, LightGBM, SVM, and KNN from the scikit-learn library [34], we assigned higher weights to minority classes and lower weights to majority classes. These weights were computed based on total samples and class distribution, resulting in the class weights of 3.36 for AKI and 0.59 for non-AKI, and the class weights of 3.07 for AKD and 0.60 for non-AKD. Importantly, this adjustment was exclusively implemented in the training set to ensure realistic evaluation in the testing phase.

### AI-driven web application

Leveraging the best-performing model, we constructed a AI-driven web application within the streamlit python-based framework to put the model into use. The application are used to predict AKI and AKD in hospitalized pediatric patients, respectively. By inputting the patient’s indicators, the probability of AKI or AKD can be determined.

### Statistical analysis

To minimize the bias caused by missing data, variables with more than 15% missing values were excluded from this study and missing data were interpolated using multiple interpolation. By employing LR to calculate the necessary sample size for assessing AKD as the outcome, we determined that a minimum of 221 patients was required to achieve 90% power to detect an effect size of 0.10.

Continuous variables with normal distribution were expressed as mean ± standard deviation (SD) and compared with independent t-test. Others violated the normality assumption were expressed as median (interquartile range) and analyzed by Mann-Whitney U test. Categorical variables were described as percentages and compared using chi-squared test or Fisher’s exact test. All statistical tests were two-sided, with a *P* value of < 0.05 indicating statistical significance. All statistical analysis were performed under Python (version 3.9.13) and Visual Studio Code (version 1.81.1) integrated development environment.

### Stability assessment

To evaluate the model’s stability, we conducted a subgroup analysis focusing on pediatric patients across different age groups. Recognizing that children undergo distinct physiological changes at various stages of growth and development, we categorized the patients into three groups: infancy (28 days to 1 year), childhood (2 to 10 years), and adolescence (11 to 18 years).

## Results

### Baseline characteristics

**Figure 1.**
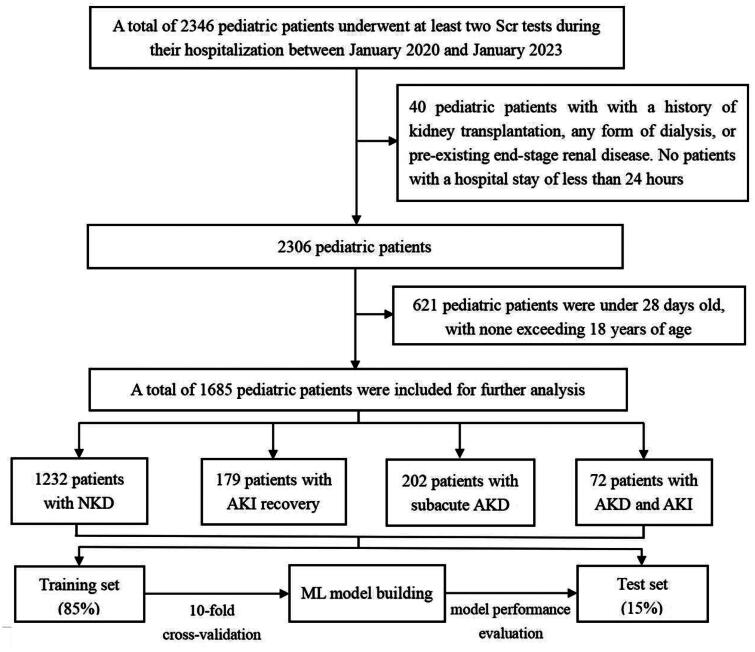
Flowchart depicting participant inclusion and exclusion criteria. Scr: serum creatinine; AKI: acute kidney injury; AKD: acute kidney disease; NKD: no kidney disease; ML: machine learning.

A total of 1,685 pediatric patients met the inclusion criteria for analysis (Figure 1). Among the 1,685 patients, no diagnoses of ‘acute kidney injury’ or ‘acute renal failure’ were documented based on ICD-10 codes at the time of admission. The incidence of AKI was 14.90% (251/1,685), while AKD occurred in 16.26% (274/1,685) of patients. The overall mortality rate for the cohort was 1.48% (25/1,685). Based on renal function trajectories, we identified the incidences of AKI recovery, subacute AKD, and AKD with AKI as 10.62% (179/1,685), 11.99% (202/1,685), and 4.27% (72/1,685), respectively. Corresponding mortality rates were 5.59% (10/179) for AKI recovery, 1.49% (3/202) for subacute AKD, and 6.94% (5/72) for AKD with AKI. Notably, AKD with AKI was associated with the highest mortality rate and extended hospital stays, indicating a more severe condition and a poorer prognosis (Table 1).

**Table 1.** Comparison of baseline characteristics between patients with normal renal function and those with renal injury.

Features	Total	NKD	Acute/subacute renal impairment				*P*-value
			AKI recovery	subacute AKD	AKD with AKI	Total	
Number	1,685	1,232 (73.12%)	179 (10.62%)	202 (11.99%)	72 (4.27%)	453 (26.88%)	–
Demography							
Age (years)	7.28 ± 5.67	7.51 ± 5.65	6.86 ± 6.27	6.139 ± 5.11	7.708 ± 5.66	6.67 ± 5.70	0.007
Gender (male) (%)	993 (58.93)	709 (57.55)	102 (56.98)	134 (134.00)	48 (66.67)	284 (62.69)	0.057
Heart rate (bpm)	94.15 ± 13.75	93.21 ± 13.08	98.72 ± 16.69	94.17 ± 10.83	98.75 ± 19.93	96.70 ± 15.14	<0.001
Laboratory test							
WBC (×10^9^/L)	10.56 ± 24.45	9.47 ± 20.91	14.13 ± 27.30	9.55 ± 19.28	23.21 ± 58.86	13.53 ± 32.02	0.012
Neutrophils counts(×10^9^/L)	4.41 ± 5.92	4.04 ± 5.71	6.37 ± 6.46	3.53 ± 4.01	8.27 ± 9.31	5.40 ± 6.36	<0.001
Prothrombin time (sec)	11.57 ± 3.45	11.33 ± 2.48	12.24 ± 5.80	12.00 ± 4.22	12.88 ± 5.98	12.23 ± 5.19	<0.001
Fibrinogen (g/L)	2.76 ± 1.06	2.81 ± 1.04	2.68 ± 1.10	2.54 ± 0.97	2.68 ± 1.31	2.62 ± 1.08	0.001
PT% (%)	112.18 ± 28.69	114.32 ± 28.20	105.48 ± 28.23	109.98 ± 28.54	98.49 ± 32.20	106.37 ± 29.24	<0.001
Scr (μmol/L)	55.04 ± 43.27	55.80 ± 28.05	42.84 ± 33.00	52.90 ± 23.01	78.39 ± 159.97	52.97 ± 69.48	0.400
eGFR (ml/min/1.73m^2^)	157.76 ± 42.11	153.69 ± 37.30	182.63 ± 61.98	158.74 ± 32.90	162.93 ± 57.92	168.85 ± 51.46	<0.001
Serum sodium (mmol/L)	139.32 ± 4.79	139.67 ± 3.50	137.53 ± 10.30	139.01 ± 3.32	138.50 ± 4.80	138.35 ± 7.13	<0.001
Blood glucose (mmol/L)	5.02 ± 2.17	4.87 ± 1.39	6.16 ± 4.94	4.81 ± 1.57	5.46 ± 2.39	5.45 ± 3.46	0.001
LDH (U/L)	364.11 ± 567.67	336.74 ± 522.80	387.40 ± 480.93	410.08 ± 564.25	645.67 ± 1149.98	438.57 ± 669.80	0.004
Total protein (g/L)	61.13 ± 9.04	61.81 ± 8.72	60.46 ± 10.13	58.68 ± 9.13	58.19 ± 9.49	59.30 ± 9.62	<0.001
Albumin (g/L)	36.69 ± 6.85	36.94 ± 6.57	36.41 ± 7.51	36.15 ± 7.61	34.76 ± 7.24	36.03 ± 7.52	0.024
Complications							
Respiratory failure (N,%)	46 (2.73)	23 (1.87)	10 (5.59)	10 (4.95)	3 (4.17)	23 (5.08)	<0.001
Diabetes (N,%)	12 (0.71)	1 (0.08)	11 (6.15)	0	0	11 (2.43)	<0.001
Shock (N,%)	33 (1.96)	16 (1.30)	7 (3.91)	5 (2.48)	5 (6.94)	17 (3.75)	0.001
MODS (N,%)	14 (0.83)	3 (0.24)	9 (5.03)	1 (0.50)	1 (1.39)	11 (2.43)	<0.001
Medication							
PPI (N,%)	978 (58.04)	680 (55.19)	82 (45.81)	156 (77.23)	60 (83.33)	298 (65.78)	<0.001
ACEI/ARB (N,%)	532 (31.57)	348 (28.25)	65 (36.31)	75 (37.13)	44 (61.11)	184 (40.62)	<0.001
Diuretics (N,%)	873 (51.81)	596 (48.38)	90 (50.28)	129 (63.86)	58 (80.56)	277 (61.15)	<0.001
Antibiotics (N,%)	1,149 (68.19)	809 (65.67)	120 (67.04)	156 (77.23)	64 (88.89)	340 (75.06)	<0.001
Hypnotics and sedatives (N,%)	611 (36.26)	401 (32.55)	94 (52.51)	70 (34.65)	46 (63.89)	210 (46.36)	<0.001
Heparin (N,%)	500 (29.67)	330 (26.79)	63 (35.20)	66 (32.67)	41 (56.94)	170 (37.53)	<0.001
Aspirin (N,%)	37 (22.14)	265 (21.51)	38 (21.23)	39 (19.31)	31 (43.06)	108 (23.84)	0.307
Adverse outcome							
Length of Stay (days)	23.27 ± 14.28	23.73 ± 14.53	16.51 ± 11.10	25.09 ± 13.03	27.07 ± 15.53	22.02 ± 13.50	0.029
Mortality (N,%)	25 (1.48)	7 (0.57)	10 (5.59)	3 (1.49)	5 (6.94)	18 (3.97)	<0.001

NKD: no kidney disease; AKI: acute kidney injury; AKD: acute kidney disease; WBC: white blood cell count; PT%: prothrombin time percentage; Scr: serum creatinine; eGFR: estimated glomerular filtration rate; LDH: lactate dehydrogenase; MODS: multiple organ dysfunction syndrome; PPI: proton pump inhibitor; ACEI/ARB: angiotensin-converting enzyme inhibitor/angiotensin receptor blocker.

The median age of patients was 7.28 ± 5.67 years, with 58.93% being male. Patients with kidney injury were younger, averaging 6.67 ± 5.70 years compared to 7.51 ± 5.65 years for those with normal renal function. Additionally, conditions such as respiratory failure, diabetes, and shock were significantly more common in the kidney injury group (*p* < 0.05). Medications also differed between the groups, with patients in the kidney injury group more frequently receiving PPI, ACE inhibitors/angiotensin receptor blockers (ACEI/ARB), hypnotics, sedatives, and diuretics (Table 1). Baseline clinical data comparing the AKI and non-AKI groups are provided in Supplement Table 1.

### Model performance

**Figure 2.**
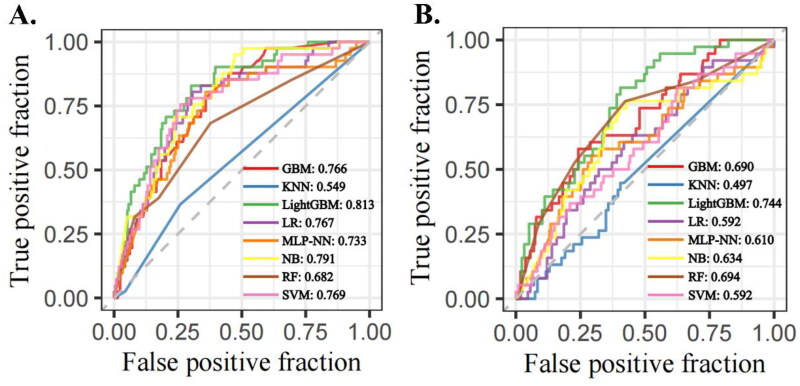
ROC curves for eight machine learning models to predict AKI or AKD. (A) AKI predictions; (B) AKD predictions. GBM: Gradient Boosting Machine; KNN: K-Nearest Neighbors; LightGBM: Light Gradient Boosting Machine; LR: Logistic regression; MLP-NN: Multilayer Perceptron Neural Network; NB: Naive Bayes classifier; RF: Random Forest; SVM: Support Vector Machines.

Eight machine learning models were trained on the training set and evaluated on the test set. For AKI prediction, the LightGBM model achieved the highest performance with an AUROC of 0.813, followed by the NB model with an AUROC of 0.791. Similarly, for AKD prediction, LightGBM had the top AUROC of 0.744, while the RF model had an AUROC of 0.694 (Figure 2). In addition to excelling in AUROC, LightGBM also showed high accuracy rates for both AKI and AKD predictions, at 0.719 and 0.636, respectively. It also achieved the highest F1 scores across all models evaluated, demonstrating a well-balanced performance between precision and recall (Supplement Table 2).

In the voting ensemble model that combines eight base machine learning models, the AUROC for AKI prediction was 0.803, and for AKD prediction was 0.733 (Supplement Figure 1). Both lower than the standalone LightGBM model. For the stacking ensemble model using different meta-models, the ensemble with RF as the meta-model achieved the highest performance in AKI prediction, while the ensemble with LR as the meta-model performed best in AKD prediction, with AUROCs of 0.808 and 0.747, respectively (Supplement Table 3).

Given that the LightGBM model demonstrated optimal performance in AKI prediction and that, in AKD prediction, the stacking ensemble model’s AUROC only exceeded the LightGBM model by 0.03 (0.747 vs. 0.744) while incurring a higher computational cost, we selected the LightGBM model as the preferred choice for further visualization and analysis. Decision curve analysis (DCA) was used to illustrate the net benefit of the LightGBM model across various probability thresholds, offering deeper insight into its practical utility in clinical decision-making (Supplement Figure 2). The DCA results highlighted conditions where the LightGBM model provides a significant net benefit, underscoring its potential as an effective tool for the early identification of AKI and AKD in pediatric patients.

### Model interpretations

**Figure 3.**
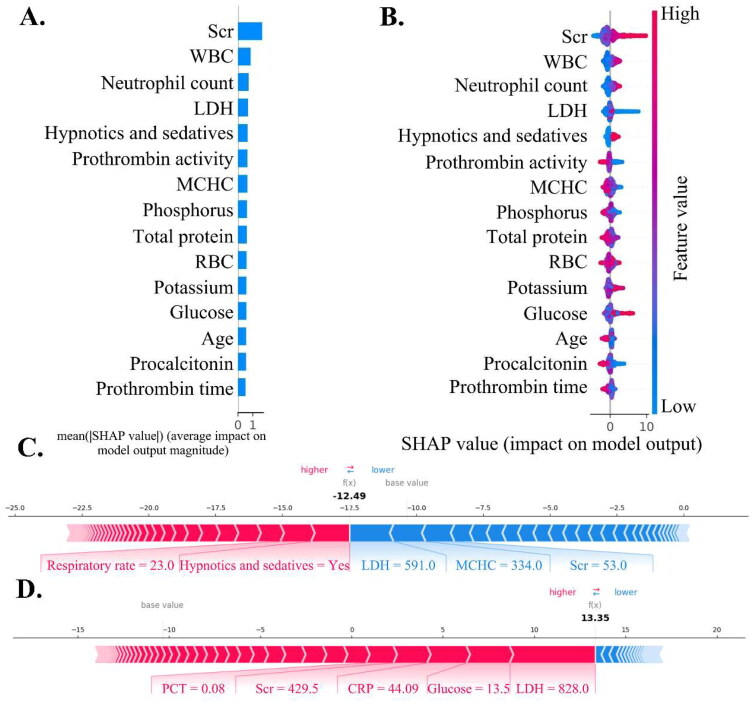
Global and individual prediction of AKI patients. (A) and (B) illustrate summary and beeswarm plots for LightGBM models predicting AKI, highlighting the top 15 significant features. In (A), bar length represents the SHAP value’s absolute size, indicating its contribution to predictions. In (B) each dot represents a patient; dots further right on the horizontal axis denote higher positive SHAP values, while those further left denote higher negative SHAP values, with dot colors reflecting observed feature values. (C) and (D) show force plots for personalized AKI risk prediction in two patients: Patient C’s risk is under 10%, while patient D’s exceeds 90%. the base value averages predicted results. A red right arrow indicates supportive measures for AKI prediction, whereas a blue left arrow signifies negative impact. Values below represent patients’ actual characteristics, with arrow lengths depicting their impact on predictions. Scr: serum creatinine; WBC: white blood cell count; LDH: lactate dehydrogenase; MCHC: mean corpuscular hemoglobin concentration; RBC: red blood cell count; PCT: procalcitonin; CRP: C-reactive protein.

**Figure 4.**
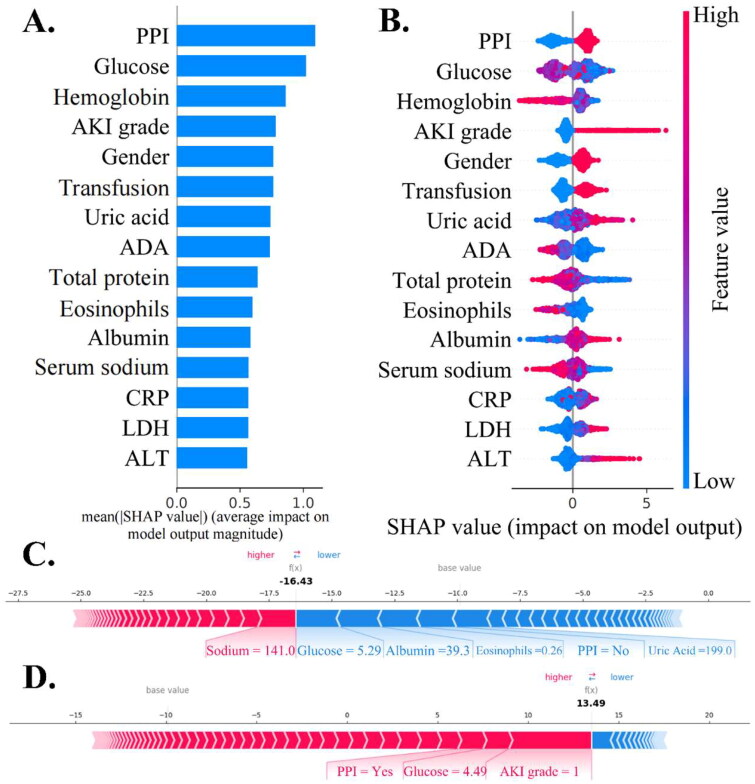
Global and individual prediction of AKD patients. (A) and (B) depict the summary and beeswarm plots respectively for LightGBM models predicting AKD, showcasing the top 15 significant features related to AKD prediction. (C) and (D) display force plots of the LightGBM model for personalized AKD risk prediction in two patients. The predicted risk of AKD in patient C is less than 10%, while the risk of AKD in patient D is more than 90%. PPI: proton pump inhibitor; AKI grade: acute kidney injury grade; ADA: adenosine deaminase; CRP: C-reactive protein; LDH: lactate dehydrogenase; ALT: alanine transaminase.

The SHAP analysis identified the top five features for predicting AKI as serum creatinine (Scr) levels, white blood cell (WBC) count, neutrophil count, lactate dehydrogenase (LDH) levels, and the use of hypnotics and sedatives (Figure 3(A-B)). For AKD prediction, SHAP highlighted the top five predictors as PPI use, blood glucose level, hemoglobin level, AKI grade, and gender (Figure 4(A-B)). SHAP force plots (Figures 3(C-D) and 4(C-D)) and decision plots (Supplement Figure 3) illustrated individualized predictions for single patients, providing a clear visual of how different features influenced each prediction.

For example, two patients both had over a 90% probability of developing AKI (Figure 3(D), Supplement Figure 3A). Although their predicted outcomes were similar, the primary contributing predictors varied. For the first patient, the top features were LDH, blood glucose, C-reactive protein (CRP), Scr, and procalcitonin (PCT) (Figure 3(D)). In contrast, for the second patient, the main predictors were anion gap, heart rate, WBC count, platelet count (PLT), and total protein level (Supplement Figure 3A). This variability in feature importance emphasizes the necessity of personalized risk assessment, as predictor significance can differ between individual and global interpretations.

SHAP interaction plots provided a visual representation of correlations among the top 15 features (Supplement Figure 4), while dependence plots highlighted two indicators with notable associations (Supplement Figure 5). These plots showed how changes in one variable’s values influenced the model’s output *via* changes in another variable. For instance, patients under 10 years showed higher SHAP values, suggesting worse renal outcomes, especially when treated with hypnotics and sedatives. Additionally, AKI grade was a significant predictor for AKD, with stages 2-3 yielding higher SHAP values than stage 1. Elevated uric acid levels also contributed to AKD prediction, with SHAP values increasing notably when uric acid levels exceeded 200 µmol/L, and even more so above 400 µmol/L, thus aiding in risk stratification and management (Supplement Figure 5).

### Web application

The final predictive model has been integrated into a web application for clinician use. To maintain efficiency and simplicity, it was necessary to reduce the input variables on the prediction page appropriately. When the model was constructed using only the top five predictors, its performance decreased. Therefore, the final version of the online prediction tool was developed using the top ten features, resulting in an online risk calculator for predicting AKI and AKD in hospitalized pediatric patients (Table 2). Clinicians can input patient characteristics and press ‘predict’ to estimate the risk of AKI or AKD. The web application is accessible at https://pediatricsakiakdapp-ynpcegzrsv4csf3ymweect.streamlit.app/capable online.

**Table 2.** LightGBM performance metrics to predict AKI and AKD on the test set.

Target	AUROC	Precision	Recall	Accuracy	F1 score	MCC	Brier score
AKI prediction							
Top 5 features	0.684	0.299	0.561	0.696	0.390	0.248	0.167
Top 10 features	0.743	0.285	0.902	0.664	0.433	0.342	0.167
Top 15 features	0.776	0.337	0.780	0.715	0.471	0.372	0.160
Top 20 features	0.799	0.422	0.659	0.747	0.514	0.410	0.150
All features	0.813	0.412	0.683	0.719	0.514	0.412	0.144
AKD prediction							
Top 5 features	0.546	0.167	0.974	0.613	0.285	0.145	0.187
Top 10 features	0.640	0.253	0.526	0.636	0.342	0.216	0.181
Top 15 features	0.725	0.313	0.526	0.680	0.392	0.303	0.150
Top 20 features	0.735	0.338	0.711	0.747	0.458	0.357	0.148
All features	0.744	0.263	0.816	0.636	0.397	0.302	0.140

LightGBM: Light Gradient Boosting Machine; AKI: acute kidney injury; AKD: acute kidney disease; AUROC: area under the receiver operating characteristic curve; MCC: Matthews correlation coefficient.

### Subgroup analysis

The final LightGBM model exhibited stable performance across different age groups. Specifically, the model achieved its best performance for predicting AKI in the adolescent group, with an AUROC of 0.750. In predicting AKD, the infant group had the highest performance, with an AUROC of 0.723 (Supplement Figure 6).

## Discussion

In this retrospective study, we found that among 1685 general pediatric inpatients, the incidence rates of AKI and AKD were 14.90% and 16.26%, respectively. The mortality rate for children with acute and subacute kidney injury was significantly higher than that for children without kidney injury, at 3.97% compared to 0.57%. We evaluated eight machine learning algorithms and two ensemble methods to build prediction models, with the LightGBM model demonstrating exceptional performance (AUROC: 0.813 and 0.744). The ensemble algorithms also demonstrated strong performance, achieving AUROC values of 0.808 and 0.747 in predicting AKI and AKD, respectively. Interpreting machine learning models and visualizing predictions remains challenging. we applied SHAP to improve interpretability and mitigate the ‘black box’ issue [35]. Ultimately, we developed an AKI and AKD risk prediction model for hospitalized children based on the LightGBM algorithm, implementing it for clinical application using Streamlit.

To our knowledge, this is the first AKI risk assessment tool specifically designed for general pediatric inpatients. Luo et al. utilized preoperative and intraoperative variables to construct an XGBoost model for predicting AKI risk related to pediatric cardiac surgery [13], while Hu developed an RF prediction model for patients in pediatric intensive care units [15]. In contrast to previous AKI studies, which primarily focused on high-risk pediatric populations in intensive care or post-cardiac surgery settings [13,14], this study encompasses a broader patient population, allowing greater attention to previously overlooked pediatric groups. Additionally, this is the first predictive model for AKD in pediatric patients, enabling the timely identification of children at high risk for AKD, as AKD independently predicts poor renal prognosis and CKD progression [36].

Although the renal function trajectory of AKI-AKD has been studied in adults [37], further investigation is required in pediatric patients due to differences in etiology, risk factors, and mechanisms between children and adults [5]. This study is the first to examine the impact of renal function trajectory on adverse outcomes in pediatric patients. We found that the AKD with AKI had the highest mortality rate (6.94%), while subacute AKD had the lowest (1.49%). We speculate that hospitalized patients with kidney injury lasting more than 7 days, particularly those following the AKD with AKI pattern, are more likely to experience acute adverse outcomes, such as mortality. In contrast, subacute AKD appears to be more closely associated with chronic renal deterioration in pediatric patients, likely due to its stronger association with nephrotoxic drugs [6]. For example, PPI use ranks as a top predictor of AKD.

The role of PPI in kidney injury has been widely discussed. The mechanism by which PPI cause kidney injury is linked to acute interstitial nephritis [38]. A self-controlled case series identified PPI use as an independent risk factor for AKI [39]. Xie et al. further confirmed that, without AKI mediation and intervention, individuals exposed to PPI had an increased risk of adverse chronic renal outcomes, including CKD, CKD exacerbation, and progression to end-stage renal disease [40]. However, these findings have been limited to adults. Our study found that PPI use in pediatric patients is strongly associated with kidney injury within 7 to 90 days, ranking first in SHAP importance and indicating an increased risk of CKD development. This may be due to the underdeveloped and more susceptible nature of children’s kidneys to drugs and toxins [41]. Therefore, medication use in pediatric patients requires particular caution.

The effect of age on AKI in pediatric patients is closely linked to the heightened renal susceptibility in children. Our SHAP dependence plot indicated that patients under 10 years old exhibited higher SHAP values for AKI prediction. Moreover, the use of hypnotic and sedative drugs in children under 5 years old intensified the age-related risk of AKI, underscoring the adverse effects of drug nephrotoxicity on kidney development and renal function. This risk stratification is essential for managing kidney injury in pediatric patients, highlighting the need to prioritize monitoring of nephrotoxic medications in children younger than 10 years.

The occurrence of AKD in pediatric patients largely depends on the severity of AKI occurrence, consistent with findings in adult studies [42]. Children’s immature immune systems make them more susceptible to infections than adults [43]. Our findings show that inflammatory cells, such as WBC and neutrophils, are key predictors of AKI in pediatric patients, ranking among the top five predictors according to SHAP analysis. These cells contribute to kidney damage through endothelial adhesion and cytokine release, primarily regulating acute inflammation within the first 24 hours [44]. Additionally, childhood malnutrition can suppress immunity and alter renal hemodynamics [45]. Studies have shown that hypoalbuminemia is an independent predictor of AKI and post-AKI mortality in adults [46], a finding consistent with our results in pediatric patients. Low hemoglobin levels predispose patients to renal hypoxia and oxidative stress [47], with Damien et al. demonstrated that hemoglobin levels and anemia are independently associated with kidney damage [48], aligning with our model’s predictions for hospitalized pediatric AKD.

This study has several limitations. First, as a single-center study, it lacks pediatric patient data from other institutions, limiting the model’s stability and generalizability. Future evaluations in diverse populations are needed. Second, due to the subjective nature of urine volume data collection, elevated urine volume was not used as a diagnostic criterion for acute and subacute kidney injury, which may have led to the omission of some cases. Finally, the short follow-up period prevented analysis of CKD progression within the AKI-AKD-CKD continuum. Future research should examine long-term renal outcomes in pediatric patients with acute and subacute kidney injury.

## Conclusion

The incidence of AKI and AKD in hospitalized children is significant and warrants attention. The renal function trajectories of different AKI-AKD types are closely associated with varying degrees of adverse prognoses, meriting further investigation. Machine learning-based risk assessment models for AKI and AKD have demonstrated strong performance, and the corresponding web application facilitates practical implementation. This tool assists in the timely identification of high-risk patients, ultimately improving the prognosis for pediatric patients.

## Supplementary Material

Supplemental Material

## Data Availability

Due to the presence of sensitive information from human participants, the dataset and code used in this study are not publicly available. However, they can be requested from the corresponding author under reasonable conditions.
