# Supplementary material for: Acute kidney disease in hospitalized pediatric patients: risk prediction based on an artificial intelligence approach
Source: Ren Fail. 2024 Dec 12;46(2):2438858. doi: 10.1080/0886022X.2024.2438858 (PMC11648138; doi:10.1080/0886022X.2024.2438858)
Supplement: Supplemental Material [file IRNF_A_2438858_SM0155.docx]

**Supplementary Materials**


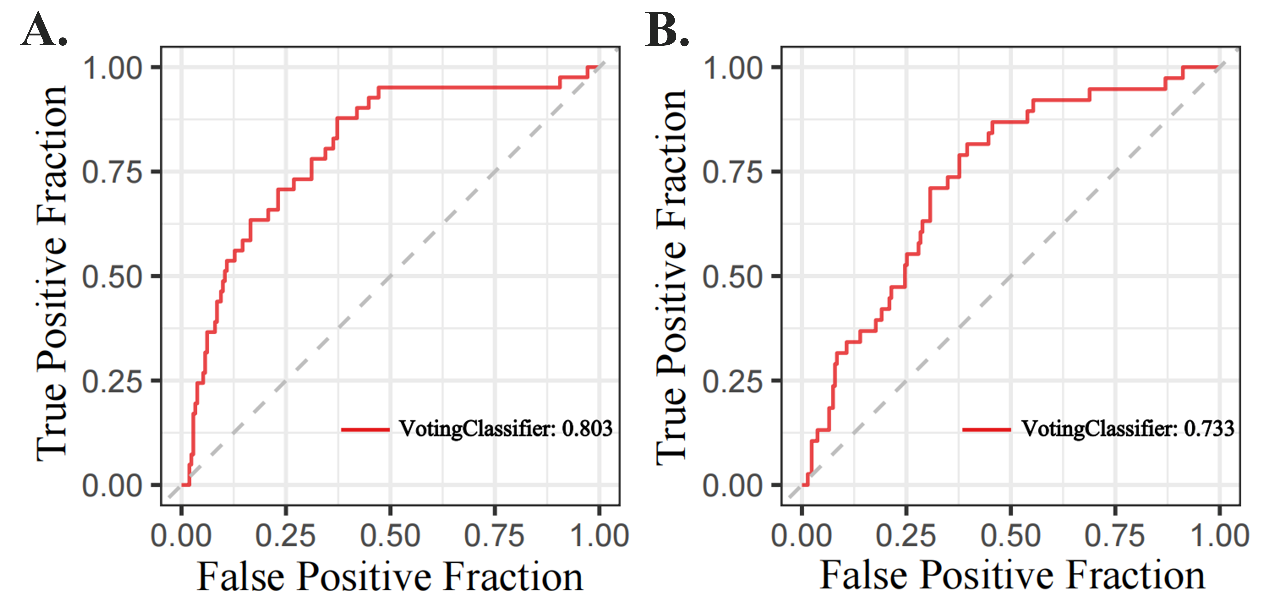


**Supplement Figure 1.** The voting ensemble learning model predicts the performance of two outcomes: (A) AKI prediction and (B) AKD prediction.


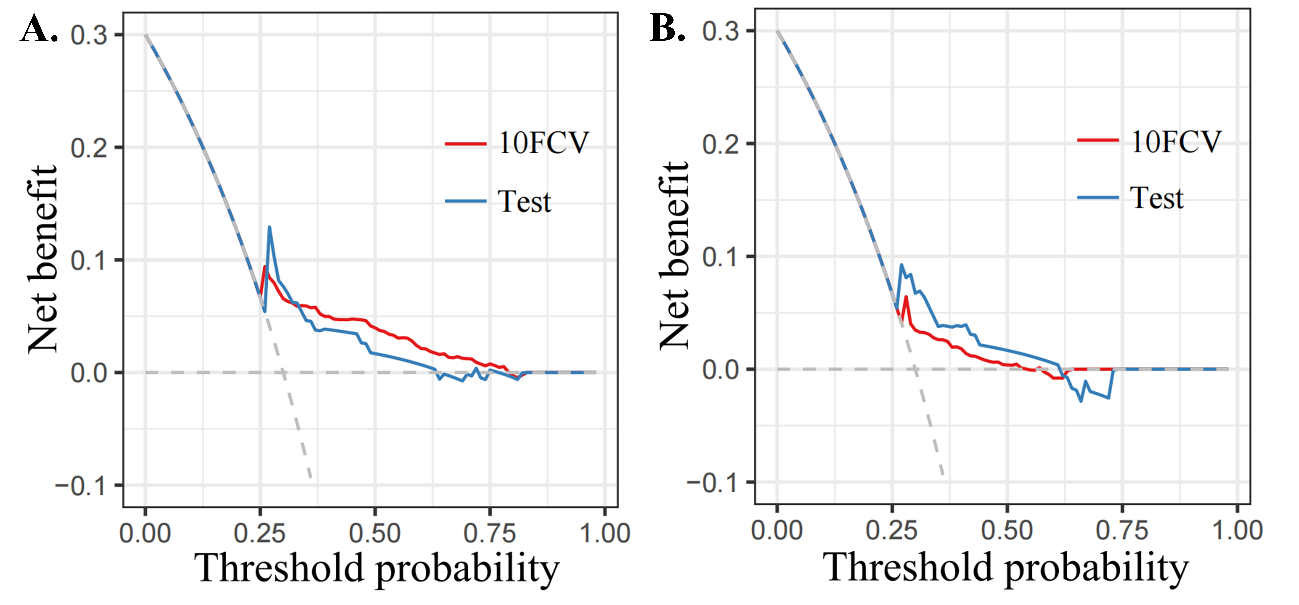


**Supplement Figure 2.** DCA plots for AKI and AKD prediction in pediatic patients. A shows the AKI prediction and B shows the AKD prediction. The horizontal coordinate represents the Threshold probability that is used to make a treatment decision. For example, when the threshold is 0.25, it means that treatment is taken when the predicted probability of a patient becoming ill exceeds 25%. The ordinate represents the Net benefit of the model at different thresholds. The dashed gray line represents the baseline at which it is assumed that all patients are not receiving treatment. The red and blue lines represent the model's performance on the ten-fold cross-validation and test sets, respectively. When the red or blue line is above the zero line, it means that the model has a net benefit under the corresponding threshold relative to no treatment or all treatment. 10FCV: 10-fold cross-validation.


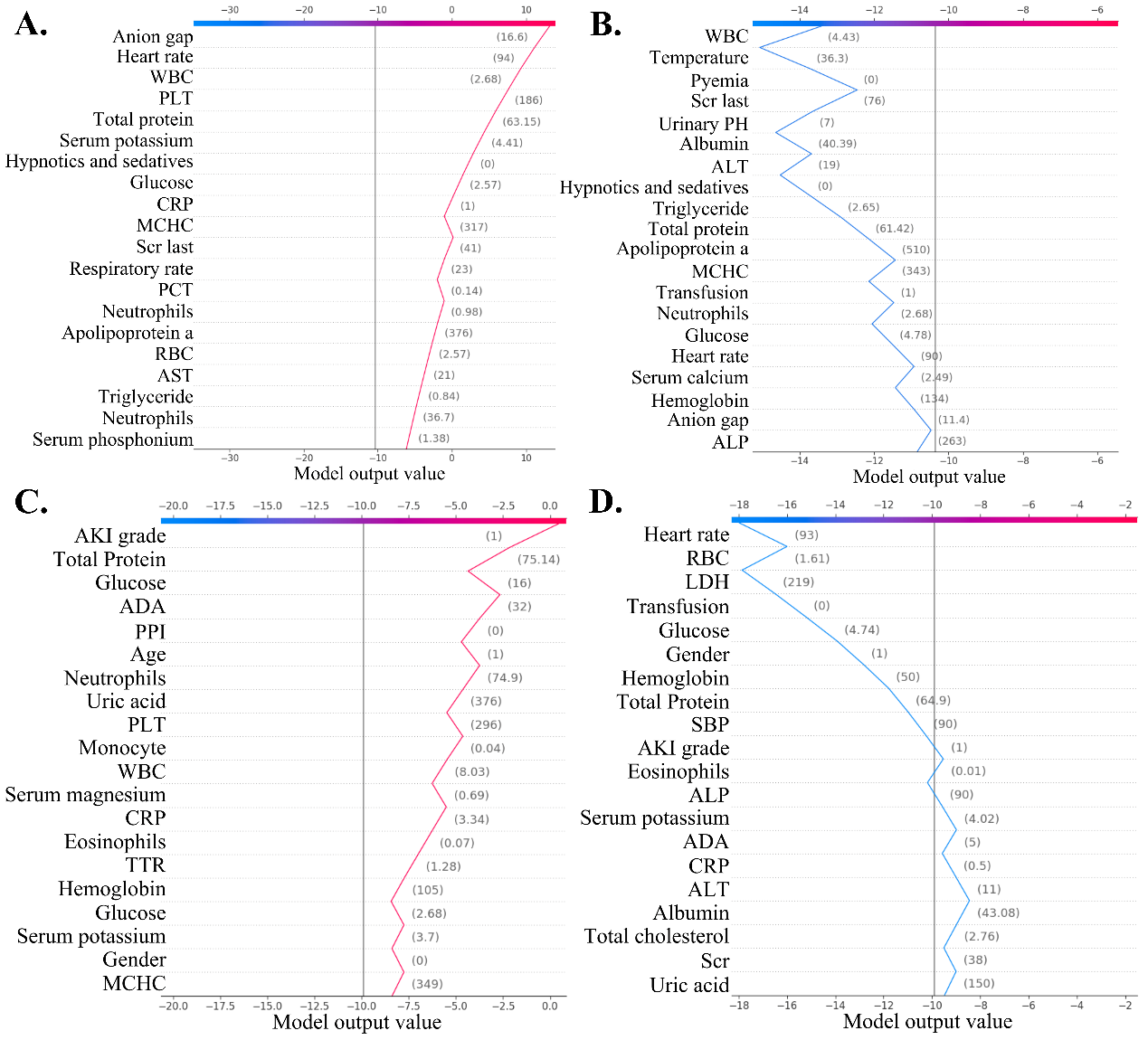
**Supplement Figure 3.** Decision plots illustrate the individualized prediction process for four patients. A and B show the process for predicting AKI outcomes, while patients C and D show the AKD outcomes. According to the model prediction, the probability of predicting AKI or AKD is greater than 90% for patients A and C, and less than 10% for patients B and D. The horizontal axis represents the SHAP value, and the influence of features on individual prediction increases from bottom to top on the vertical axis. The top color bar indicates the SHAP value magnitude: red denote a positive prediction, and blue denote a negative prediction. WBC: White blood cell; PLT: Platelet; CRP: C-reactive protein; MCHC: Mean corpuscular hemoglobin concentration; Scr last: Serum creatinine last; PCT: Procalcitonin; RBC: Red blood cell; AST: Aspartate aminotransferase; ALT: Alanine aminotransferase; ALP: Alkaline phosphatase; AKI grade: Acute kidney injury grade; ADA: Adenosine deaminase; PPI: Proton pump inhibitor; TTR: Thrombin time ratio; LDH: Lactate dehydrogenase; SBP: Systolic blood pressure.


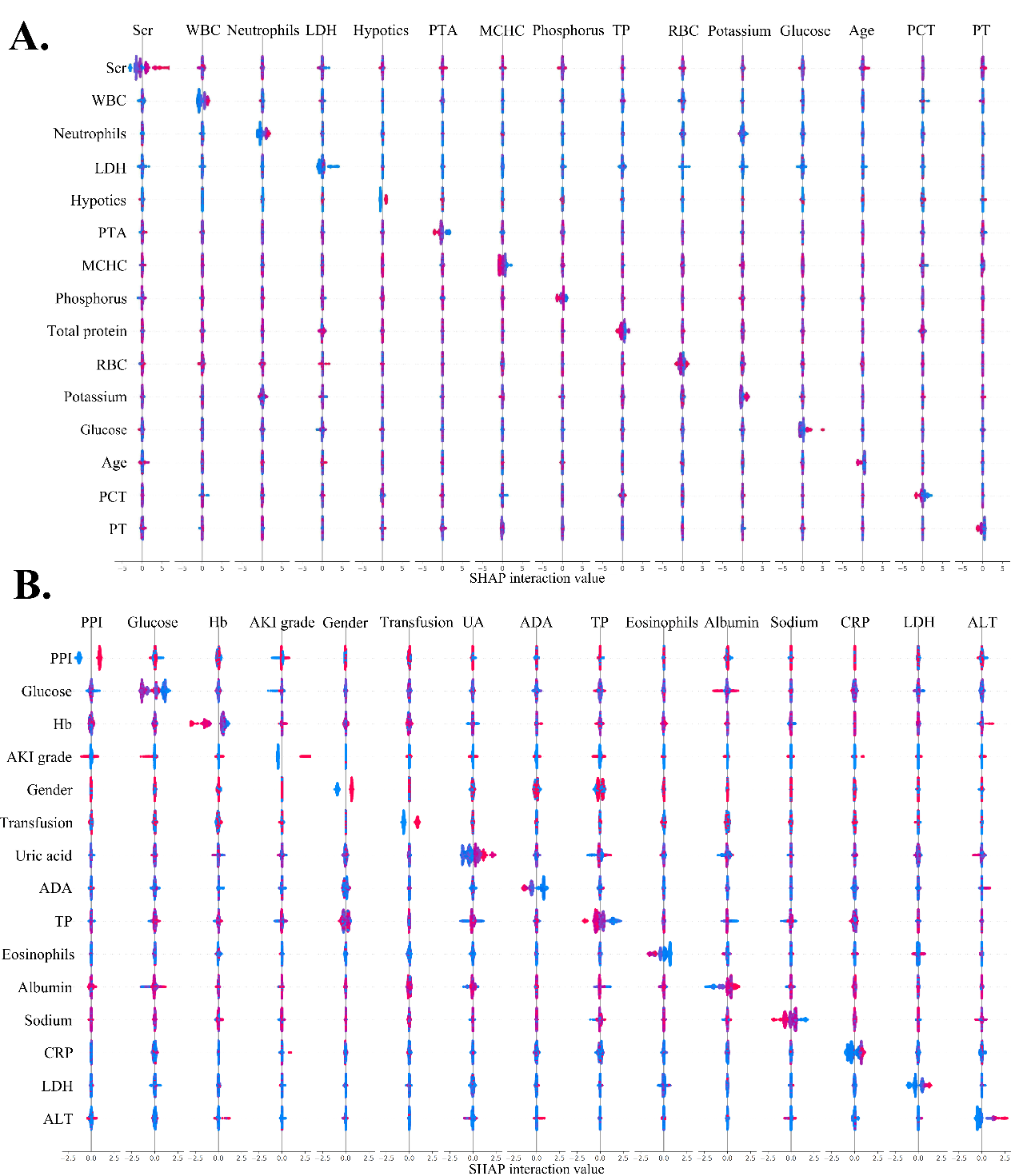


**Supplement Figure 4.** Interaction plots for AKI and AKD prediction. A displays interactions between the top 15 features for AKI, and B displays interactions for AKD. The interaction plots estimate the impact of each feature on the model’s predictions when interacting with other features. A larger absolute SHAP value at the interaction point signifies a stronger interaction between the corresponding features and a greater impact on the prediction. Red indicates a positive SHAP interaction value, increasing the model’s predicted output, while blue decreases the predicted output. Scr: Serum creatinine; WBC: White blood cell; LDH: lactate dehydrogenase; PTA: Prothrombin time activity; MCHC: Mean corpuscular hemoglobin concentration; RBC: Red blood cell; PCT: Procalcitonin; PT: Prothrombin time; PPI: Proton pump inhibitor; Hb: Hemoglobin; AKI grade: Acute kidney injury grade; ADA: Adenosine deaminase; TP: Total protein; CRP: C-reactive protein; ALT: Alanine aminotransferase.


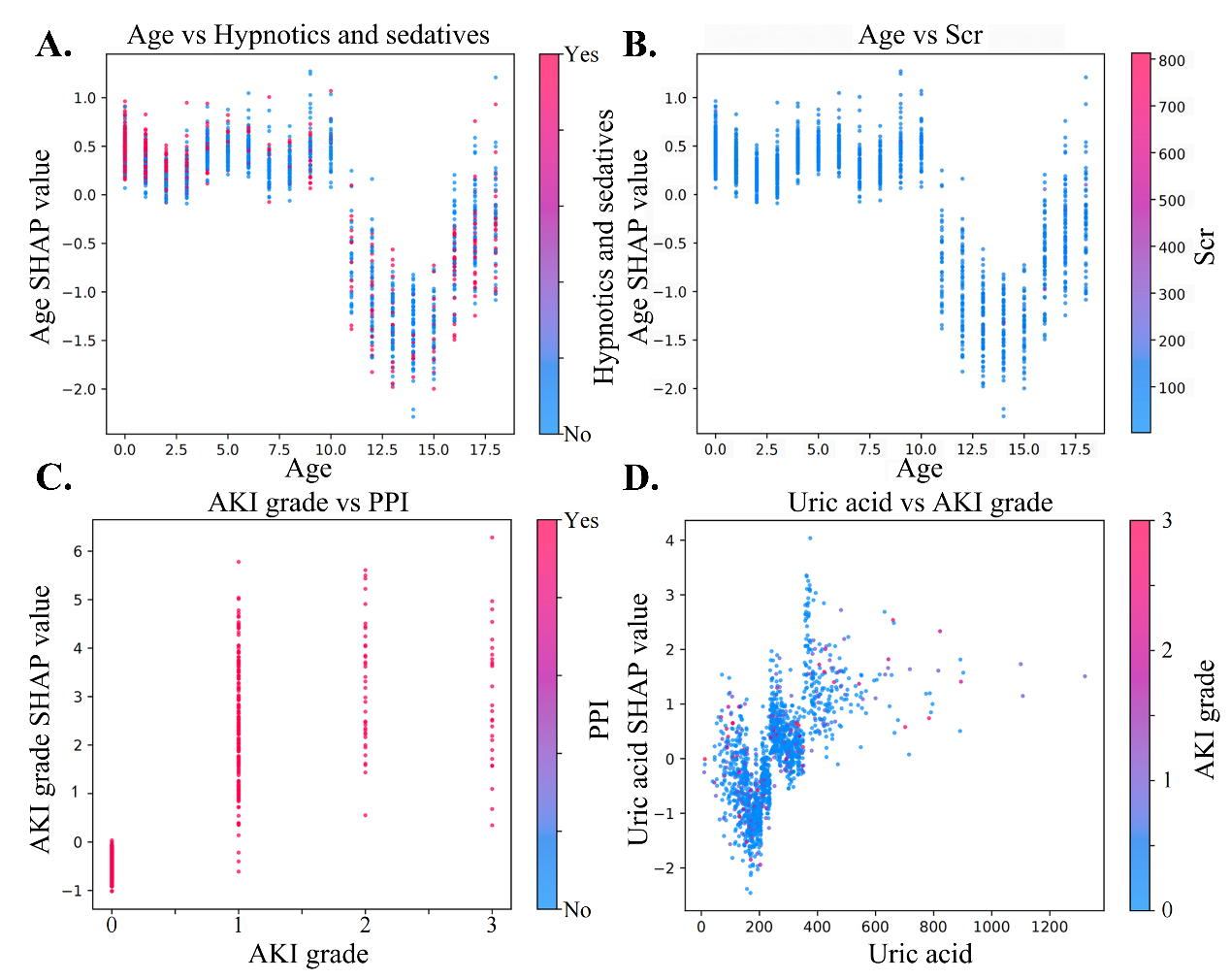
 **Supplement Figure 5.** Dependence plots predicting AKI and AKD. A and B show SHAP dependence plots for predicting AKI variables. C and D display dependence plots for predicting AKD variables. A illustrates the relationship between age and the use of hypnotic and sedative drugs. B shows the relationship between age and weight. C depicts the relationship between AKI grade and PPI. D demonstrates the relationship between UA levels and AKI grade. Each dot represents a patient; the horizontal axis represents a variable, the vertical axis represents the SHAP value for that variable, and the color strip on the right indicates the condition of another variable for that patient. SHAP: SHapley Additive exPlanations; Scr: Serum creatinine; AKI grade: Acute kidney injury grade; PPI: Proton pump inhibitor.


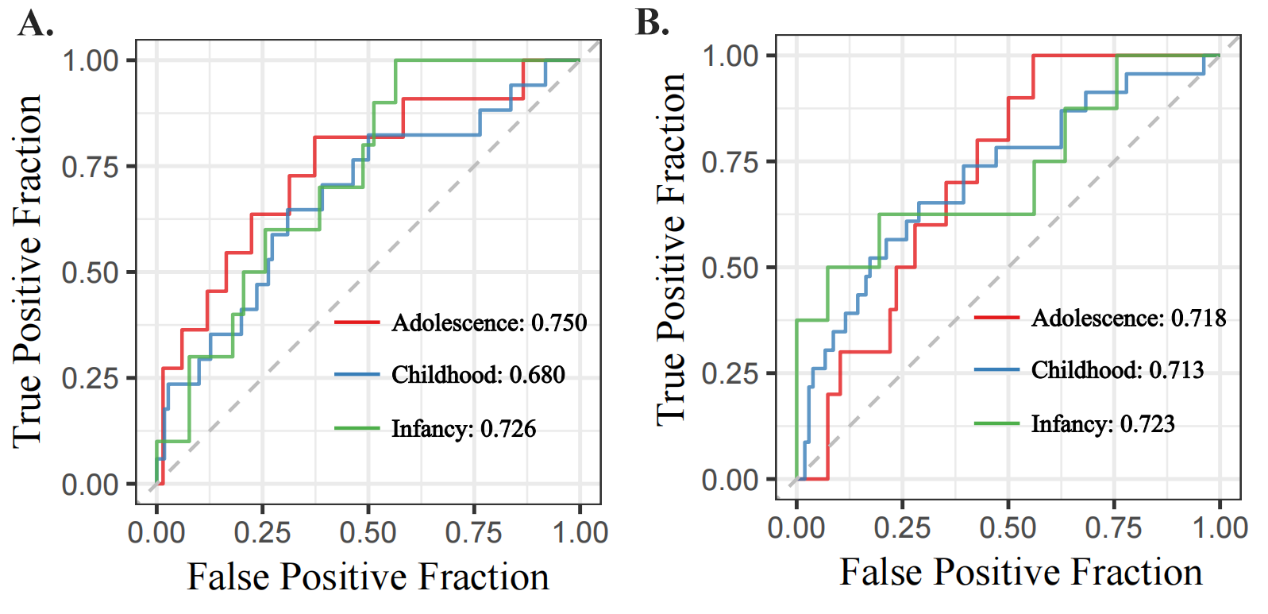


**Supplement Figure 6.** ROC curves for the LightGBM model across different age groups. A shows predictions for AKI, and B shows predictions for AKD. The age groups are defined as follows: infancy (28 days to 1 year), childhood (2 to 10 years), and adolescence (11 to 18 years).

**Supplement Table 1. Comparison of baseline characteristics between patients in the non-AKI group and the AKI group**

| **Features** | **Total** | **Non-AKI** | **AKI** | ***P-value*** |
| --- | --- | --- | --- | --- |
| Number | 1685 | 1434 (85.10%) | 251 (14.90%) | - |
| Age (years) | 7.28 ± 5.67 | 7.32 ± 5.60 | 7.10 ± 6.10 | 0.607 |
| Male (%) | 993 (58.93) | 843 (58.79) | 150 (59.76) | 0.772 |
| Blood transfusion (%) | 725 (43.03) | 659 (45.96) | 66 (26.29) | <0.001 |
| Surgical history (%) | 244(14.48) | 216 (15.06) | 28 (11.16) | 0.105 |
| Surgery (%) | 345(20.47) | 275 (19.18) | 70 (27.89) | 0.002 |
| Respiratory rate (bpm) | 22.74 ± 3.87 | 22.62 ± 3.73 | 23.39 ± 4.56 | 0.012 |
| Heart rate (bpm) | 94.15 ± 13.75 | 93.35 ± 12.79 | 98.73 ± 17.64 | <0.001 |
| RBC (×10^12^/L) | 3.70 ± 0.93 | 3.67 ± 0.90 | 3.85 ± 1.07 | 0.007 |
| WBC (×10^9^/L) | 10.56 ± 24.45 | 9.48 ± 20.68 | 16.73 ± 39.14 | 0.004 |
| Neutrophil counts (×10^9^/L) | 4.41 ± 5.92 | 3.97 ± 5.50 | 6.91 ± 7.42 | <0.001 |
| Hemoglobin (g/L) | 0.83 ± 2.08 | 0.76 ± 1.80 | 1.26 ± 3.19 | 0.015 |
| Monocyte counts (×10^9^/L) | 105.41 ± 25.94 | 104.77 ± 24.93 | 109.04 ± 30.89 | 0.039 |
| NEUT% (%) | 47.54 ± 24.42 | 46.36 ± 24.06 | 54.30 ± 25.42 | <0.001 |
| HCT (%) | 31.73 ± 7.57 | 31.56 ± 7.27 | 32.65 ± 9.04 | 0.074 |
| CRP (mg/L) | 15.01 ± 30.53 | 14.60 ± 30.12 | 17.35 ± 32.71 | 0.188 |
| Prothrombin time (sec) | 11.57 ± 3.45 | 11.42 ± 2.80 | 12.42 ± 5.85 | 0.008 |
| PT% (%) | 112.18 ± 28.69 | 113.71 ± 28.28 | 103.47 ± 29.53 | <0.001 |
| eGFR (ml/min/1.73m^2^) | 157.76 ± 42.11 | 154.40 ± 36.75 | 176.98 ± 61.38 | <0.001 |
| Serum sodium (mmol/L) | 139.32 ± 4.79 | 139.58 ± 3.48 | 137.81 ± 9.07 | 0.003 |
| Serum chloride (mmol/L) | 103.64 ± 5.03 | 103.88 ± 4.20 | 102.28 ± 8.17 | 0.003 |
| Phosphorus (mmol/L) | 1.49 ± 0.38 | 1.51 ± 0.37 | 1.39 ± 0.45 | <0.001 |
| Blood glucose (mmol/L) | 5.02 ± 2.17 | 4.86 ± 1.42 | 5.96 ± 4.37 | <0.001 |
| ALT (U/L) | 84.99 ± 527.49 | 69.46 ± 251.74 | 173.68 ± 1225.46 | 0.181 |
| AST (U/L) | 88.53 ± 661.80 | 65.42 ± 226.05 | 220.59 ± 1623.81 | 0.132 |
| Total bilirubin (μmol/L) | 17.74 ± 37.02 | 17.08 ± 35.41 | 21.55 ± 45.03 | 0.136 |
| LDH (U/L) | 364.11 ± 567.67 | 347.07 ± 529.25 | 461.49 ± 744.28 | 0.020 |
| Total protein (g/L) | 61.13 ± 9.04 | 61.37 ± 8.84 | 59.81 ± 9.99 | 0.012 |
| Albumin (g/L) | 36.69 ± 6.85 | 36.83 ± 6.73 | 35.94 ± 7.46 | 0.078 |
| Urine protein |  |  |  | 0.198 |
| Negative (%) | 1600 (94.96) | 1369 (95.47) | 231 (92.03) |  |
| 1+ (%) | 25 (1.48) | 19 (1.32) | 6 (2.39) |  |
| 2+ (%) | 25 (1.48) | 19 (1.32) | 6 (2.39) |  |
| 3+ (%) | 35 (2.08) | 27 (1.88) | 8 (3.19) |  |
| Urine glucose |  |  |  | <0.001 |
| Negative (%) | 1597 (94.78) | 1375 (95.89) | 222 (88.45) |  |
| 1+ (%) | 24 (1.42) | 16 (1.12) | 8 (3.19) |  |
| 2+ (%) | 20 (1.19) | 16 (1.12) | 4 (1.59) |  |
| 3+ (%) | 23 (1.36) | 16 (1.12) | 7 (2.79) |  |
| 4+ (%) | 21 (1.25) | 11 (0.77) | 10 (3.98) |  |
| Respiratory failure (N,%) | 46(2.73) | 33 (2.30) | 13 (5.18) | 0.010 |
| Diabetes (N,%) | 12 (0.71) | 1 (0.07) | 11 (4.38) | <0.001 |
| Shock (N,%) | 33 (1.96) | 21 (1.46) | 12 (4.78) | 0.001 |
| MODS (N,%) | 14 (0.83) | 4 (0.28) | 10 (3.98) | <0.001 |
| PPI (N,%) | 978(58.04) | 836 (58.30) | 142 (56.57) | 0.609 |
| ACEI/ARB (N,%) | 532(31.57) | 423 (29.50) | 109 (43.43) | <0.001 |
| Diuretics (N,%) | 160(9.50) | 129 (9.00) | 31 (12.35) | 0.094 |
| CCB (N,%) | 87(5.16) | 71 (4.95) | 16 (6.37) | 0.347 |
| Cardiac glycosides (N,%) | 84(4.99) | 64 (4.46) | 20 (7.97) | 0.019 |
| Adrenergic drugs (N,%) | 326(19.35) | 252 (17.57) | 74 (29.48) | <0.001 |
| Antibiotics (N,%) | 1149(68.19) | 965 (67.29) | 184 (73.31) | 0.059 |
| Alkylating agent (N,%) | 219(13.00) | 199 (13.88) | 20 (7.97) | 0.010 |
| Metabolic antagonists (N,%) | 370 (21.96) | 338 (23.57) | 32 (12.75) | <0.001 |
| Alkaloid (N,%) | 329 (19.53) | 302 (21.06) | 27 (10.76) | <0.001 |
| Hypnotics and sedatives drugs (N,%) | 611 (36.26) | 471 (32.85) | 140 (55.78) | <0.001 |
| Heparin (N,%) | 500 (29.67) | 396 (27.62) | 104 (41.43) | <0.001 |
| Mortality (N,%) | 25(1.48) | 10 (0.70) | 15 (5.98) | <0.001 |
| Non-AKI: Non-acute kidney injury; AKI: Acute kidney injury; RBC: Red blood cell; WBC: White blood cell; NEUT%: Neutrophil percentage; HCT: Hematocrit; CRP: C-reactive protein; PT%: Prothrombin time percentage; eGFR: Estimated glomerular filtration rate; ALT: Alanine aminotransferase; AST: Aspartate aminotransferase; LDH: Lactate dehydrogenase; MODS: Multiple organ dysfunction syndrome; PPI: Proton pump inhibitor; ACEI/ARB: Angiotensin-converting enzyme inhibitor/Angiotensin receptor blocker; CCB: Calcium channel blocker. | | | | |

**Supplement Table 2. The performance of eight machine learning models to predict AKI and AKD on the test set (All features)**

| **Models** | **AUROC** | **Precision** | **Recall** | **Accuracy** | **F1 score** | **MCC** | **Brier score** |
| --- | --- | --- | --- | --- | --- | --- | --- |
| **AKI prediction** | | | | | | | |
| LightGBM | 0.813 | 0.412 | 0.683 | 0.719 | 0.514 | 0.412 | 0.144 |
| GBM | 0.766 | 0.361 | 0.537 | 0.664 | 0.431 | 0.317 | 0.130 |
| RF | 0.682 | 0.259 | 0.683 | 0.632 | 0.376 | 0.270 | 0.128 |
| KNN | 0.549 | 0.162 | 1.000 | 0.680 | 0.279 | 0.088 | 0.164 |
| MLP | 0.733 | 0.303 | 0.805 | 0.668 | 0.440 | 0.332 | 0.124 |
| NB | 0.791 | 0.367 | 0.707 | 0.755 | 0.483 | 0.375 | 0.221 |
| SVM | 0.769 | 0.360 | 0.756 | 0.743 | 0.488 | 0.386 | 0.131 |
| LR | 0.767 | 0.337 | 0.805 | 0.711 | 0.475 | 0.377 | 0.121 |
| **AKD prediction** | | | | | | | |
| LightGBM | 0.744 | 0.263 | 0.816 | 0.636 | 0.397 | 0.302 | 0.140 |
| GBM | 0.690 | 0.297 | 0.579 | 0.731 | 0.393 | 0.265 | 0.123 |
| RF | 0.694 | 0.290 | 0.526 | 0.605 | 0.374 | 0.243 | 0.124 |
| KNN | 0.503 | 0.150 | 1.000 | 0.561 | 0.261 | 0.021 | 0.174 |
| MLP | 0.610 | 0.250 | 0.553 | 0.684 | 0.344 | 0.197 | 0.129 |
| NB | 0.634 | 0.246 | 0.737 | 0.621 | 0.368 | 0.242 | 0.359 |
| SVM | 0.621 | 0.236 | 0.553 | 0.664 | 0.331 | 0.177 | 0.128 |
| LR | 0.593 | 0.227 | 0.447 | 0.577 | 0.301 | 0.143 | 0.132 |
| AUROC: Area under the receiver operating characteristic curve; MCC: Matthews correlation coefficient; AKI: Acute kidney injury; AKD: Acute kidney disease; LightGBM: Light gradient boosting machine; GBM: Gradient boosting machine; RF: Random forest; KNN: K-nearest neighbors; MLP: Multi-layer perceptron; NB: Naive bayes; SVM: Support vector machine; LR: Logistic regression. | | | | | | | |

**Supplement Table 3. Stacking ensemble performance based on different meta-models on the test set.**

| Meta-model | AUROC | Precision | Recall | Accuracy | F1 Score | MCC | Brier Score |
| --- | --- | --- | --- | --- | --- | --- | --- |
| **AKI prediction** | | | | | | | |
| LR | 0.779 | 0.381 | 0.780 | 0.747 | 0.512 | 0.419 | 0.116 |
| RF | 0.808 | 0.424 | 0.683 | 0.715 | 0.523 | 0.423 | 0.111 |
| GBM | 0.786 | 0.402 | 0.805 | 0.775 | 0.537 | 0.452 | 0.115 |
| SVM | 0.520 | 0.519 | 0.341 | 0.791 | 0.412 | 0.334 | 0.129 |
| KNN | 0.676 | 0.287 | 0.610 | 0.692 | 0.391 | 0.246 | 0.139 |
| MLP-NN | 0.799 | 0.538 | 0.512 | 0.747 | 0.525 | 0.436 | 0.107 |
| NB | 0.800 | 0.378 | 0.829 | 0.751 | 0.519 | 0.435 | 0.162 |
| LightGBM | 0.728 | 0.369 | 0.585 | 0.696 | 0.453 | 0.331 | 0.164 |
| **AKD prediction** | | | | | | | |
| LR | 0.747 | 0.318 | 0.711 | 0.727 | 0.439 | 0.333 | 0.115 |
| RF | 0.720 | 0.276 | 0.711 | 0.676 | 0.397 | 0.279 | 0.121 |
| GBM | 0.698 | 0.265 | 0.579 | 0.613 | 0.364 | 0.236 | 0.126 |
| SVM | 0.580 | 0.357 | 0.395 | 0.684 | 0.375 | 0.258 | 0.128 |
| KNN | 0.577 | 0.202 | 0.526 | 0.617 | 0.292 | 0.116 | 0.167 |
| MLP-NN | 0.564 | 0.286 | 0.368 | 0.593 | 0.322 | 0.187 | 0.134 |
| NB | 0.740 | 0.313 | 0.684 | 0.676 | 0.430 | 0.323 | 0.178 |
| LightGBM | 0.665 | 0.433 | 0.342 | 0.605 | 0.382 | 0.291 | 0.159 |
| AUROC: Area under the receiver operating characteristic curve; MCC: Matthews correlation coefficient; AKI: Acute kidney injury; AKD: Acute kidney disease; LR: Logistic regression; RF: Random forest; GBM: Gradient boosting machine; SVM: Support vector machine; KNN: K-nearest neighbors; MLP-NN: Multi-layer perceptron neural network; NB: Naive bayes; LightGBM: Light gradient boosting machine. | | | | | | | |
